# Relevance of *Helicobacter pylori* infection in Egyptian multiple sclerosis patients

**DOI:** 10.1186/s41983-018-0043-x

**Published:** 2018-12-07

**Authors:** Steven Emil Gerges, Taha Kamel Alosh, Salma Hamed Khalil, Mona Mokhtar Waheed El Din

**Affiliations:** Faculty of Medicine, Ain Shams University, 19 Awad Fahmy st–El Zatoun, Cairo, Egypt

**Keywords:** Antibodies, Autoimmune, Heat shock protein 60, *Helicobacter pylori*, Multiple sclerosis

## Abstract

**Background:**

Multiple sclerosis (MS) is an autoimmune demyelinating disorder. The etiology of MS remains unknown exactly. *Helicobacter pylori* heat shock proteins were suggested as a potential trigger of immune system causing MS.

**Objectives:**

The aim of this study was to assess the level of anti-*Helicobacter pylori* heat shock proteins 60 (Hp hsp60) antibodies at patients of MS and to correlate it with various epidemiological and clinical data.

**Subjects and methods:**

This study design was a cross-sectional case control one. A total of 65 patients with multiple sclerosis diagnosed according to 2010 revised McDonald criteria and other 65 age- and sex-matched healthy controls were included in this study. All participants were subjected to full history taking, complete neurological examination including Expanded Disability Status Scale (EDSS) for the patients, measurement of serum level of anti-Hp hsp60 IgG using ELISA technique, and MRI brain for all the patients, being a goldstone for inclusion in the study.

**Results:**

There was statistically significant high level of anti-Hp hsp60 IgG at MS patients especially secondary progressive multiple sclerosis (SPMS) patients. Moreover, a positive statistically significant correlation was found between it and age of patients, duration of illness, and EDSS.

**Conclusion:**

We conclude that hsp60 of Hp may be a useful biomarker for attesting course progression in MS.

## Introduction

The exact prevalence of multiple sclerosis in Egypt cannot be given since no organized survey for this purpose was carried out and door to door study was difficult. A community-based survey using 2005 revised McDonald’s criteria for diagnosis of multiple sclerosis (MS) in Al Quseir, Egypt, has found an MS prevalence of 13.74/100,000 [1].

There is also data scarcity on the prevalence of *Helicobacter pylori* infection in an Egyptian population and the only study found was a study in a rural area of the country that revealed an overall seropositivity rate of 91.7% of this population. The rate of infection was different in different age groups with an increasing trend in older ages [2].

The etiology of MS remains unknown. Several environmental factors, including microbial agents, have been considered potential inducers of the disease [3].

Among the microbial agents, *Helicobacter pylori* (Hp) has been considered a possible infectious trigger of the disease [4]. This assumption may be supported by high incidence and prevalence of gastrointestinal symptoms at MS [5].

At the antigen level, several Hp antigens have been considered important for the loss of immunological tolerance to myelin antigens, particularly heat shock proteins (hsp) [6].

High degree of sequence homology between mammalian and pathogenic heat shock protein has been found in several studies. The immune system may recognize heat shock proteins as dominant pathogenic antigens or potentially harmful self-antigens due to its high conserved nature. Conserved epitopes of heat shock proteins among mammalian cells and prokaryotes may lead to cross reactivity and induces immune reactivity to self-heat shock proteins which eventually results in autoimmune diseases [7].

The immune reaction that occurs in MS is thought to trigger a process of neurodegenerative damage that leads to clinical signs. In this scenario, extracellular hsp, namely hsp60 and hsp70, exacerbates the immune response by acting as an adjuvant for myelin peptides and as a proinflammatory cytokines [8].

In the past, high levels IgG antibodies against hsp70 have been reported in the cerebrospinal fluid (CSF) of patients with MS. However, significant difference in the levels of antibodies against hsp27, hsp60, or hsp90 was not observed [9].

Antibody responses to Hp-specific hsp60 have not been studied in great detail in MS despite being one of the most immunogenic heat shock proteins [10].

In the present study, we assess the level of this antibody in patients with MS as a biomarker for the inflammatory processes and for possible etiological role.

## Subjects and methods

A group of 65 patients with MS from the outpatient MS clinic of Ain Shams University hospital chosen randomly and a group of 65 age- and sex-matched healthy controls were included in this study. The study was performed between July 2016 and July 2017. The procedures followed were in accordance with the ethical standards of the responsible committee on human experimentation and with the principles of Helsinki Declaration [11]. Informed consent was obtained from all participants, and ethical committee permission from our institution was obtained before starting our work. Inclusion criteria were as follows: patients known to have MS according to revised 2010 McDonald criteria for diagnosis of MS [12]. Exclusion criteria were as follows: patients with neuromylitis optica (Devic’s disease) or other demyelinating and inflammatory disorders of central nervous system (CNS), and other chronic medical disorders (diabetes mellitus, chronic kidney disease, chronic liver disease, other endocrinal disorders).

All the participants were subjected to the following: clinical evaluation including general medical history and examination, full neurological history and examination, diagnosis of definite MS according to revised 2010 McDonald criteria for diagnosis of MS [12], assessment of severity of MS in each patient by using Expanded Disability Status Scale (EDSS) [13], and quantitative assessment of the level of IgG antibodies against Hp hsp60 at the serum of both cases and controls by using enzyme-linked immunosorbent assay (ELISA) technique; the detection range of the kit was from (78.13–5000 pg/mL) and the materials and instruments were obtained from Elabscience biotechnology (www.elabscience.com). Magnetic resonance imaging (MRI) brain (all cases were subjected to MRI brain with T1 weighted with and without contrast, T2 weighted and fluid-attenuated inversion recovery (Flair) scans to apply proposed MAGNIMS criteria for diagnosis of MS [14]).

## Statistical analysis

Statistical presentation and analysis of the present study was conducted, using the mean, standard deviation, student *t* test, Chi-square, linear correlation coefficient, and analysis of variance (ANOVA) tests. Collected data in this study were analyzed using the statistical package for the social sciences (SPSS, version 17; SPSS Inc., Chicago, Illinois, USA).

Unpaired Student *T* test was used to compare between two groups in quantitative data. Chi-square test was used to compare independent row and column variables without indicating strength or direction of the relationship. Fisher’s exact test and Yates’ corrected chi-square are computed for analysis of contingency tables. Linear correlation coefficient was used for detection of correlation between two quantitative variables in one group. ANOVA test was used for comparison among different times in the same group in quantitative data.

## Results

The study included 65 patients with definite multiple sclerosis and 65 age- and sex-matched healthy individuals. The case group included 17 males (26.15%) and 48 females (73.85%) and their age ranged from 15 to 60 years with mean age of 34.723 ± 9.711 years. Forty-two patients (64.2%) were diagnosed as relapsing remitting multiple sclerosis (RRMS), 22 patients (33.85%) were diagnosed as secondary progressive multiple sclerosis (SPMS), and 1 patient (1.54%) was diagnosed as primary progressive multiple sclerosis (PPMS). The age of onset of multiple sclerosis among the cases ranged from 13 to 49 years old with the mean age of onset was 26.908 ± 7.814 years. The duration of MS among the cases ranged from 1 to 28 years with the mean duration was 14.5 years. The EDSS ranged from 2 to 7 while the median EDSS was 4.5. The control group included 20 males (30.77%) and 45 females (69.23%) and their age ranged from 18 to 38 years with mean age of 35.215 ± 9.672 years. These data are presented in Table 1.

**Table 1:** Descriptive statistics of the study

Gender	Groups						Chi-square	
	Case		Control		Total			
	*N*	%	*N*	%	*N*	%	*X* ^2^	*P* value
Male	17	26.15	20	30.77	37	28.46	0.340	0.560
Female	48	73.85	45	69.23	93	71.54		
Total	65	100.00	65	100.00	130	100.00		
Age	Groups						*T* test	
	Case			Control			*T*	*P* value
Range	18–60			15–60			−0.290	0.773
Mean ± SD	34.723 ± 9.711			35.215 ± 9.672				
Course of MS								
			*N*		%			
RRMS			42		64.62			
SPMS			22		33.85			
PPMS			1		1.54			
Total			65		100.00			
			Range		Mean ± SD			
Age of MS onset			13–49		26.908 ± 7.814			
Duration of MS			1–28		14.5			
			Range		Median			
EDSS			2–7		4.5			

*SD* standard deviation, *RRMS* relapsing remitting multiple, *SPMS* secondary progressive multiple sclerosis, *PPMS* primary progressive multiple sclerosis, *EDSS* expanded disability status scale

In our study, we found a high statistically significant difference in level of Hp hsp60 IgG in cases compared to controls (*P* = 0.001). There was no significant statistical difference between level of anti-Hp hsp60 IgG at male and female patients of MS (*P* = 0.646). As regard the course of the MS, patients with SPMS were found to have statistically significant higher levels of anti Hp hsp60 IgG compared to those with RRMS course (*P* = 0.001). These data are presented in Table 2.

**Table 2:** Comparison between cases and controls as regard level of anti-Hp hsp60 IgG and its distribution at gender and course of MS patients

Anti hsp60 IgG level (pg/ml)	Groups			*T* test	
	Case	Control		*t*	*P* value
Range	1150–5000	550–5000		3.383	0.001*
Mean ± SD	3421.538 ± 1385.999	2570.846 ± 1479.420			
Cases		Anti-hsp60 IgG level (pg/ml)		*T* test or ANOVA	
		*N*	Mean ± SD	*T* or *F*	*P* value
Gender	Male	17	3555.882 ± 1416.160	0.462	0.646
	Female	48	3373.958 ± 1387.175		
Course of MS	RRMS	42	2775.000 ± 1186.098	24.836	< 0.001*
	SPMS	22	4702.273 ± 709.708		
	PPMS	1	2400.000 ± 0.000		

*hsp60* heat shock protein 60, *IgG* immunoglobulin G, *SD* standard deviation, *ANOVA* analysis of variance, *RRMS* relapsing remitting multiple sclerosis, *SPMS* secondary progressive multiple sclerosis, *PPMS* primary progressive multiple sclerosis

**Fig. 1:**
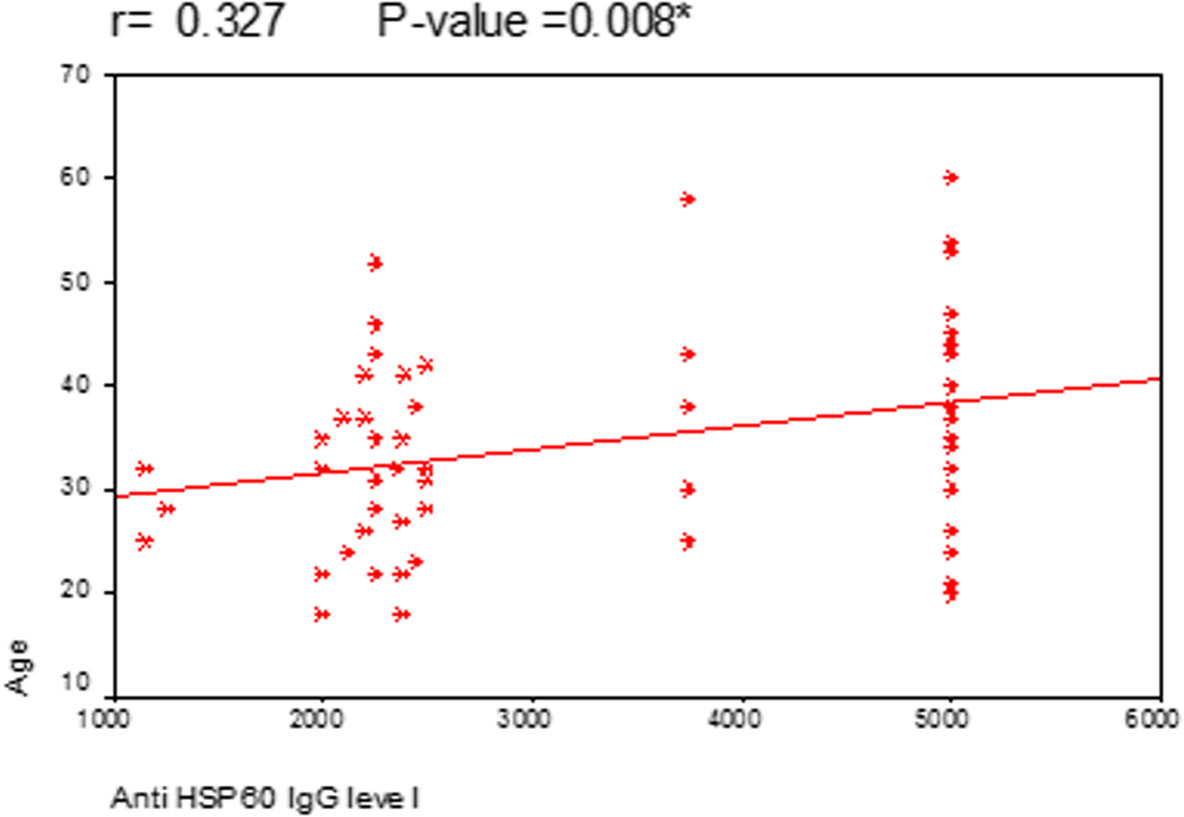
Correlation between anti Hp hsp60 IgG level and age of the MS patients

**Fig. 2:**
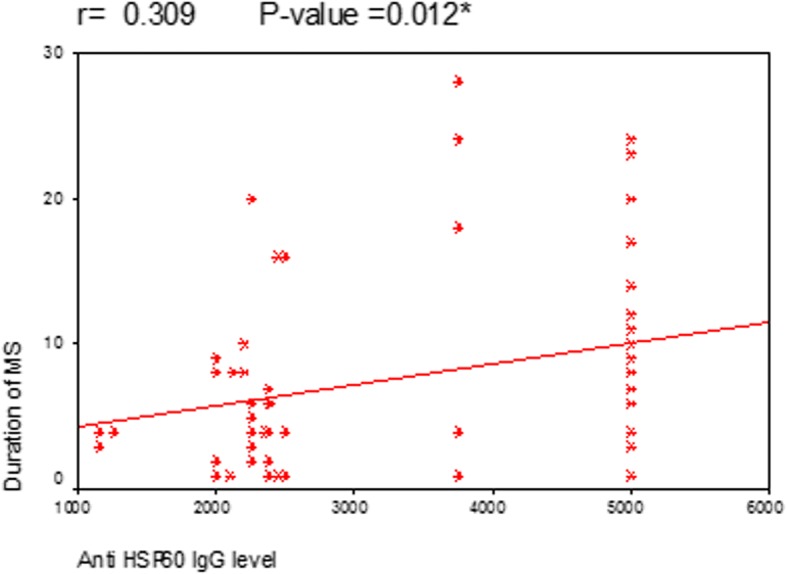
Correlation between anti Hp hsp60 IgG level and duration of MS

**Fig. 3:**
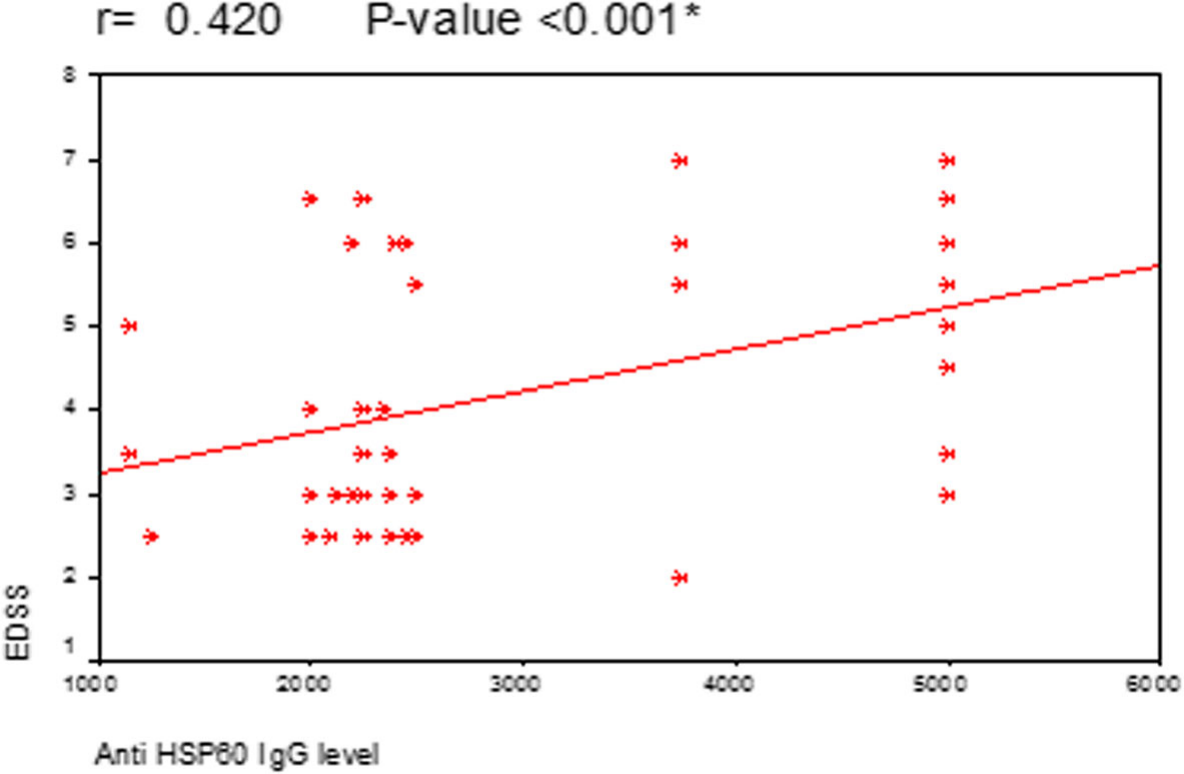
Correlation between anti Hp hsp60 IgG level and EDSS of MS patients

Correlation between age of MS patients and the level of anti-Hp hsp60 IgG showed that there is a statistically significant correlation between them (*P* = 0.008) (Fig. 1). A statistically significant correlation was also found between anti-Hp hsp60 IgG and duration of MS (*P* = 0.012) (Fig. 2). However, in both previous figures, the correlation coefficient (*r*) was 0.3 which means moderate positive relationship, even being statistically significant. This means that the high level of anti-hsp60 IgG is moderately positively correlated with the age of MS patients and duration of MS. A statistically significant strong positive correlation was found between the level of anti-hsp60 IgG and scores of EDSS (*P* = 0.001) (Fig. 3).

## Discussion

Antibody responses to Hp-specific hsp60 has not been studied in great detail in MS except at one study by Efthymiou and colleagues [15] that suggested a possible relation between infection by *H*. *pylori* species containing hsp60 antigens and triggering of MS.

These data encouraged the design of this study in order to investigate such relation and to correlate the results with various epidemiological and clinical data.

In this study, there was a significant higher level of anti Hp hsp60 IgG at MS patients compared with healthy controls, the level was especially high at SPMS patients suggesting a possible relation between infection by Hp species containing this Ag and pathogenesis of MS especially SPMS phenotype. Similar results were found by Efthymiou and colleagues [15]. This high level of reactivity of anti-Hp hsp60 at SPMS may support the recent evidence that B cells and antibodies play an important role at pathogenesis of progressive forms of MS and that compartmentalized CNS inflammation is one of the driving processes behind neurodegeneration and progression at MS; such theory is consolidated by identification of meningeal ectopic B cell follicles in SPMS patients and by successful use of B cell depleting therapy in PPMS patients [16]. Such findings may be also related to the genotype of different populations and how it affects the predisposition to MS and reaction to different antigens; a study by Atya and colleagues showed that interleukin 17 F (IL-17 F) CT genotype and C allele may be associated with a susceptibility to MS in Egyptian population by a gender-dependent mechanism that contributes to unique predisposition in females [17]. Another Egyptian study showed that signal transducer and activator of transcription 4 (STAT4) polymorphism was significantly associated with multiple sclerosis in Egyptian population [18]. Such relation between genotype and serum level of various antibodies need to be further studied.

A recent study by Lechner and collegues found a significant higher level of Hsp70 at serum of patients with RRMS and CIS than patients with SPMS and claimed that it can be used as a marker of inflammation at MS [19]. This difference in results may be related to the difference in nature between the two types of heat shock proteins or the difference between antigens and antibodies.

In this study, there was also a significant correlation between age of the MS patients, duration of the illness, and EDSS of the patients on one the hand and the level of anti-Hp hsp60 IgG on the other hand but it was not of high statistical significance. Similar results were also found by Efthymiou and colleagues [15] for age of the patients and duration of illness but not for EDSS. Such findings may reflect the tendency of longstanding inflammation to elicit antibody responses to an expanded range of targets [20] but whether this is just an epiphenomenon or has a real contributing role at disease progression is a question need to be further assessed. Lechner and colleagues found no relation between Hsp70 level and age of MS patients [19].

In this study, no significant relation was found between gender of MS patients or the age of onset of the illness and the titer of anti-Hp hsp60 IgG; Efthymiou and colleagues [15] found a positive correlation with the age of onset but not for the gender, also Lechner and collegues found no relation between gender and Hsp70 level at MS patients [19].

Limitations of this study include the relatively small number of participants especially of SPMS patients, no correlation between gastrointestinal (GIT) symptoms and results. Also, no correlation of the results with the degree of patients’ compliance with MS treatments. Moreover, the high financial cost of laboratory analysis of such antibodies at MS patients may be a barrier to its actual application as a marker for progression at disease.

## Conclusion

We conclude from this study that high anti Hp hsp60 IgG level is correlated to MS patients especially SPMS. It can be used as a biomarker for progression of such disease, being significantly correlated to age and duration of illness in a moderate level and EDSS scores to a strong level.

## Recommendations

There is a need for this study to be conducted with a larger number of participants, the relation between heat shock proteins of various species and pathogenesis of MS need to be further studied; there is a need for further studies investigating the role of Hp hsp60 specific T cell immune responses in MS patients which could clarify whether their presence in sera of MS patients is just an epiphenomenon or they play a real role at induction of the disease at genetically susceptible individuals; there is a need for further studies assessing the level of this antibody at sera of MS patients at relapse to detect if they play a role at disease exacerbation or not, and correlation between GIT symptoms and level of this antibody also needs to be studied. Also, the degree of compliance with MS medications and how it affects the level of those antibodies need to be further researched.

## Abbreviations

CNS: Central nervous system
CSF: Cerebrospinal fluid
EDSS: Expanded Disability Status Scale
ELISA: Enzyme-linked immunosorbent assay
Et al: and others
FLAIR: Fluid attenuated inversion recovery
GIT: Gastrointestinal
Hp: *Helicobacter pylori*
HSPs: Heat shock proteins
Ig: Immuno globulin
IL: Interleukin
MAGNIMUS: Magnetic resonance imaging in MS
MRI: Magnetic resonance imaging
MS: Multiple sclerosis
OD: Optical density
PG: Picogram
PPMS: Primary progressive multiple sclerosis
RRMS: Relapsing remitting multiple sclerosis
SPMS: Secondary progressive multiple sclerosis
STAT4: Signal transducer and activator of transcription 4
